# Pathogenic Characteristics of a Porcine Astrovirus Strain Isolated in China

**DOI:** 10.3390/v11121156

**Published:** 2019-12-13

**Authors:** Qingli Fang, Cui Wang, Huan Liu, Qingping Wu, Siying Liang, Minli Cen, Qinting Dong, Yingyi Wei, Ying Chen, Kang Ouyang, Zuzhang Wei, Weijian Huang

**Affiliations:** College of Animal Science and Technology, Guangxi University, No.100 Daxue Road, Nanning 530004, China; macfano@163.com (Q.F.); W1903089944@163.com (C.W.); liuchujie@yeah.net (H.L.); qingpingwu221@163.com (Q.W.); lsy1329575238@163.com (S.L.); CML123567@163.com (M.C.); dongqinting@gmail.com (Q.D.); weiyingyi99@163.com (Y.W.); yingchen@gxu.edu.cn (Y.C.); ouyangkang@gxu.edu.cn (K.O.)

**Keywords:** porcine astrovirus, pathogenesis, diarrhea, innate immunity

## Abstract

Astroviral infection is considered to be one of the causes of mammalian diarrheal diseases. It has been shown that astrovirus infections cause varying degrees of diarrhea in turkeys and mice. However, the pathogenesis of porcine astrovirus is unknown. In this study, the virulence of a cytopathic porcine astrovirus (PAstV) strain (PAstV1-GX1) isolated from the PK-15 cell line was tested using seven-day-old nursing piglets. The results showed that PAstV1-GX1 infection could cause mild diarrhea, growth retardation, and damage of the villi of the small intestinal mucosa. However, all the above symptoms could be restored within 7 to 10days post inoculation (dpi). To evaluate the innate immunity response of PAstV in vivo, the alteration of inflammatory cytokine expression in piglets infected with PAstV1-GX1 was determined using quantitative real-time reverse transcription polymerase chain reaction (qRT-PCR). The mRNA expression levels of the IFNβ and ISG54 were found to be significantly elevated in virus-infected piglets. In contrast, expression of IFNλ was downregulated in piglets infected with PAstV1-GX1. In addition, the mRNA expression of the tight junction protein 1 and 2 and zonula occludin 1, which are associated with the intestinal barrier permeability, were affected after PAstV1 infection. The present study adds to our understanding of the pathogenic mechanism of PAstV and has established an animal model for further study of pig astrovirus infection.

## 1. Introduction

Porcine astrovirus (PAstV) is single-stranded positive sense, non-enveloped RNA virus with a diameter of about 28–30 nm [1]. PAstV belongs to the family *Astroviridae*, genera *Mamastrovirus* [2], and was first discovered in 1980 [3]. Based on the phylogenetic analysis of the amino acid sequences of ORF2, PAstVs are divided into five genotypes (PAstV1–PAstV5) [4,5,6,7]. The genome length of PAstV is 6.4–7.3 kb and it contains three continuous open reading frames (ORFs), named ORF1a, ORF1b, and ORF2. ORF1a and ORF1b are situated at the 5′-end of the genome and they encode a nonstructural protein (nsp) named nsp1ab, which encompasses viral nonstructural serine protease and RNA polymerase, respectively. ORF2 is located at the 3′-end of the genome and encodes the viral capsid protein. In addition, the genome also contains a 5′untranslated region (UTR), a 3′UTR, and a poly-A tail [8].

Astroviruses (AstVs) were first identified in fecal diarrhea of children by electron microscopy in 1975 [9]. The family *Astroviridae* is divided into genera *Mamastrovirus* and *Avastrovirus*, which contain at least 19 and 3 species, respectively [10]. AstVs infections were considered to be species specific [11,12]. However, this species specificity appeared to have been breached because of the close genetic characterization seen between different species of AstVs [13,14]. Other research papers identified a single species of AstVs, which were detected in different host animals, suggesting the species barrier of AstVs may have been crossed [15,16].

Traditionally, the characteristics of AstV infection were that it was diarrheal or asymptomatic in human and other animals [17]. However, the symptoms would be more severe in immunocompromised individuals, such as those hosts who were immunosuppressive or infantile [10]. The pathogenesis of PAstV infections is still obscure and generally thought to be associated with diarrhea in piglets [6]. PAstV is usually detected in feces, suggesting that it is colonized in the intestines [18,19,20]. However, recently studies found that PAstV infection causes viremia [21] and viral encephalitis [22,23]. Compared with the *Mamastrovirus*, the *Avastrovirus* species has stronger pathogenicity. It has been shown that the turkey astrovirus type 2 can cause poultry enteritis mortality syndrome (PEMS) [24]. Duck astroviruses have been shown to be associated with duck viral hepatitis (DVH) [25]. Many studies have reported that some AstV strains, such as the human astrovirus MLB and VA strains, bovine astrovirus NeuroS1 strain, PAstV type 3, and mink astrovirus SMS strain, can damage the central nervous system (CNS) and cause encephalitis and meningitis [22,26,27,28,29], suggesting the potential for the extraintestinal and neuropathology infection processes of astroviruses.

Because of the lack of a suitable cell culture system and animal models for astroviruses, the pathogenesis of these viruses is still unclear. In this study, we carried out pathogenesis experiments involving a PAstV strain, PAstV1-GX1 (GenBank: KF787112), in 7-day-old piglets. This is the first study to focus on the pathogenicity of PAstV and provide a new animal method for understanding the mechanism of astroviral pathogenesis

## 2. Materials and Methods

### 2.1. Viruses and Cells

PK15 cells were grown in Dulbecco’s modified Eagle’s medium (DMEM) supplemented with antibiotics (100 units/mL of penicillin and 100 units/mL of streptomycin) and 10% fetal bovine serum (FBS) (Gibco). PAstV type 1 strain, named as PAstV1-GX1 (GenBank accession: KF787112), was isolated from a PAstV positive fecal sample from a diarrheal pig in a farm in Nanning, Guangxi province [30]. The PAstV1-GX1 strain was stably passaged in PK-15 cells and cultured in DMEM containing 0.5 μg/mL tosylphenylalanine chloromethyl ketone (TPCK)-treated trypsin. The low passaged viruses (P8) were used for the animal study.

### 2.2. Animals

Twenty-four seven-day-old suckling piglets of both sexes from commercial farms which, were negative for PAstV, porcine epidemic diarrhea virus (PEDV), transmissible gastroenteritis virus (TGEV), and porcine rotavirus (PRoV), were used in this study. Piglets were also free of porcine reproductive and respiratory syndrome virus (PRRSV) antibodies and pseudorabies virus (PRV) Ig antibodies. Piglets were weighed every morning before feeding, fed creep feed twice a day, and given water ad libitum.

Piglets were randomly divided into either a control group (*n* = 12) or a PAstV-challenged group (*n* = 12) and housed in individual rooms. The animals in the PAstV-challenged group were inoculated with 10 mL doses of 1×104.5 TCID50/mL by orogastric gavage at 0 days post inoculation (dpi). The control group was inoculated with 10 mL of virus-free DMEM cell culture media. Three pigs from the control and challenged groups were randomly selected for necropsy on 2, 4, 7, and 10 dpi. At necropsy, three samples were taken from the brain, spleen, lungs, kidneys, and different segments of intestinal and mesenteric lymph nodes were taken. One piece of each sample was ground and stored at −80 °C for detection of viral load and cytokine expression by quantitative real-time reverse transcription polymerase chain reaction (qRT-PCR), one piece of each sample was fixed with 10% neutral formalin for paraffin sectioning and HE staining, and the third one was fixed with 4% diethyl pyrocarbonate (DEPC)-containing paraformaldehyde for immunohistochemistry. All experimental designs were performed strictly in accordance with the recommendations in the Guide for the Care and Use of Laboratory Animals of the Ministry of Health, China, and approved by the Ethics Committee of Animal Experiments of Guangxi University (protocol number: GXU2018-044, permission data: 2 November 2018). All surgeries were performed under sodium pentobarbital anesthesia and every effort was made to minimize suffering.

### 2.3. Clinical and Necropsy Assessment

The bodyweight and temperature of each pig were recorded before feeding every day throughout the experiment. The clinical indicators included appetite status (reduction or abolition), mental condition, coat condition (soft and shiny or messy), and stool consistency (normal or watery sample). The clinical assessments were scored according to the following criteria: normal = 0, slight change = 2, obvious change = 4, large change = 6.

At necropsy, the lesions within the intestinal segments (duodenum, jejunum, ileum, cecum, colon, rectum, and mesenteric lymph nodes) and other tissues (liver, spleen, lungs, kidneys, brain, and stomach) were scored according to the following criteria: normal = 0, slight change = 2, obvious change = 4, large change = 6.

### 2.4. Histopathology and Morphometry

After 48 h post fixation in neutral buffered formalin, tissue sections were trimmed, processed, and embedded in paraffin. Then, 4 μm sections were cut and fixed on microscope slides, stained with hematoxylin and eosin (H&E), then analyzed for histopathological changes. In intestinal sections, three sections of full-length villi and crypts were measured based on tissue orientation from each of the four serial sections using the MShot image analysis system (Nikon camera, Tokyo, Japan). The mean villous length and crypt depth from each intestinal segment was used to determine statistical differences. Villous height to crypt depth ratios were also determined using the calculated means.

### 2.5. qRT-PCR

Total RNA was extracted from the tissue and feces samples by using the E.Z.N.A.^®^ HP Total RNA Kit (OMEGA biotech, Doraville, GA, USA). Then, 500 ng of RNA extraction products from each sample was reverse transcribed using the SuperScript III First-Strand Synthesis Kit (TaKaRa, Dalian, China) according to the manufacturer’s instructions. Real-time PCR was conducted with 2 μL of each cDNA sample in a total 20 uL volume using TB GreenTM premix DimerEraesrTM (TaKaRa, Dalian, China). The primers used in this study are listed in Table S1. Here, qRT-PCR was performed using the LightCycler^®^ 96 real-time system (Roche, Basel, Switzerland). The PCR reaction condition was 95 °C for 3 min, followed by 40 cycles of 95 °C for 10 s, 55 °C for 30 s, 72 °C for 30 s, and a final elongation step at 72 °C for 10 min. For quantification of PAstV, a plasmid containing the ORF1b coding region of PAstV1-GX1 was built for construction of the standard curve. Each Ct value was converted into the viral gene copy number. The effects of PAstV on transcriptional activation of inflammatory cytokines and permeable proteins were also investigated by qRT-PCR. Three independent experiments were conducted in duplicate. Relative expression values were normalized using an internal β-actin control. The fold change of relative gene expression levels was calculated following the formula: 2 − (ΔCt of gene − ΔCt of β-actin). The data are the averages derived from two independently assessed samples from each piglet in the PAstV-challenged group and each sample was measured in triplicate.

### 2.6. Immunohistochemistry (IHC)

After paraformaldehyde fixation, the tissue was rinsed for 12 h in embedding boxes. The relevant steps of dehydration, transparency, waxing, embedding, and cutting were as described above. Antigen retrieval was accomplished with Ethylenediaminetetraacetic acid (EDTA) Antigen Retrieval solution (Boster Biological tech, Beijing, China) in boiling water for 20 min and then cooled to room temperature. The slides were soaked in 3% hydrogen peroxide (Solarbio, Beijing, China) for 10 min followed by three changes of distilled water. After blocking with 5% BSA buffer (Solarbio, Beijing, China) for 30 min at 37 °C, rabbit polyclonal antibody to capsid protein of PAstV1-GX1 strain was used for the detection of PAstV antigen. The antibody was diluted 1:200 in phosphate-buffered saline (PBS) containing 0.1% Tween 20 (PBST). Each slide was transferred to a 37 °C incubator for 1 h and rinsed with PBST for 3 min. Goat anti-rabbit IgG conjugated Horseradish Peroxidase (HRP) (CWBio, Beijing, China) was diluted 1:200 in PBST and added, and the slides were transferred to a 37 °C incubator for 1 h and rinsed with PBST for 3 min. Then, 3,3′N-diaminobenzidine tetrahydrochloride (DAB) substrate chromogen (CWBio, Beijing, China) was applied to the slides for 5 min, followed by a distilled water rinse for 15 min. The slides were then counterstained with hematoxylin, mounting medium was added, and they were then cover-slipped.

A semiquantitative statistic scoring of immunohistochemistry slides based on the modification of McGarty et al. [31] was used. This was calculated by multiplying the staining intensity in four gradations with the percentage of positive cells in five gradations. The criteria method was as follows: (1) the score of the percentage of positive cells: positive cells < 10% = 0, 10% < positive cells < 25% = 1, 25% < positive cells < 50% = 2, 51% < positive cells < 75% = 3, positive cells > 75% = 4; (2) for the staining intensity, 0 = no signal, 1 = mild colored (pale yellow), 2 = moderate colored (brown), 3 = deep colored (dark brown). For the final immunoreactivity score (IRS): 0–2 indicated negative (–), 2–4 indicated mildly positive (+), 4–7 indicated moderately positive (++), and scores greater than 7 were strongly positive (+++).

### 2.7. Statistical Analysis

All the values are expressed as the means ± standard deviation (SD) or standard error of the means (SEM). Viral RNA titers were transformed as log10 RNA titers from cycle threshold values (Ct values). The mRNA expression levels of cytokines and permeable proteins and the villous lengths and crypt-depths from each intestinal segment were analyzed and compared by one-way ANOVA using GraphPad Prism software. Here, *p* < 0.05 was considered statistically significant and values of *p* < 0.01 were considered extremely significant.

## 3. Results

### 3.1. Infection of Pigs with PAstV1-GX1 Results in Mild Diarrhea

**Figure 1:**
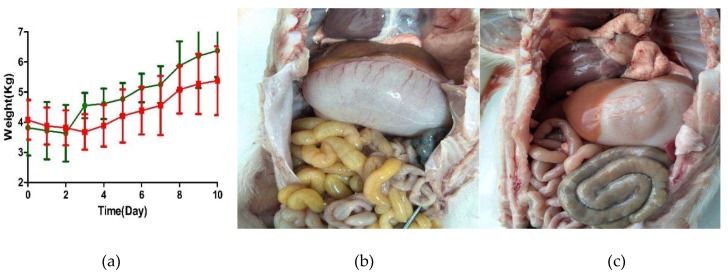
Macroscopic lesions and the mean weight changed after inoculation. (**a**) The mean weight was determined from the PAstV-challenged group (red) and control group (green) at each day after inoculation. Mean ± SD (error bars) weights are shown. Macroscopic observations of the intestine of representative piglets from (**b**) the PAstV-challenged group and from (**c**) the control group.

Both the control and PAstV-challenged groups of pigs were lively, showed no clinical symptoms, and had normal fecal shapes before inoculation. PAstV-challenged pigs displayed decreased appetite, irregular hair coat, and had semisolid feces at 2 dpi. Most of the clinical assessments were recovered after 6 dpi. There was no significant difference in body temperature between the two groups throughout the experimental period. Mild weight loss occurred at one or two days after intragastric administration in both PAstV-challenged and control pigs. PAstV-challenged pigs had a significant reduction (*p* < 0.01) in their average daily weight gain (ADWG) when compared to controls during the first week after inoculation. The ADWG of PAstV-challenged and control pigs were 0.120 kg and 0.357 kg per day, respectively. From 8 dpi to 10 dpi, the ADWG of the PAstV-challenged group returned to normal levels. However, mean total body weight remained lower in the challenged group when compared to the control group (Figure 1a).

Compared to the control group, bloat and a large amount of yellow foamy liquid in the duodenum, jejunum, and ileum was observed in PAstV-challenged piglets. Mesenteric hemorrhage was observed at 2 dpi in PAstV-challenged pigs, as shown in Figure 1b,c. The severity of lesions intensified at 4 dpi, including gaseous distention, capillary congestion in mesenteries and gastric mucosa, serious bloat, and large amounts of yellow foamy liquid in the intestine were observed, especially in the jejunum. Most of these macroscopic lesions were recovered at 7 dpi but there was still some foamy liquid in the jejunum. However, all the lesions recovered at 10 dpi. No discernible lesions appeared in the rectum, spleen, lungs, or kidneys of PAstV-challenged pigs for the duration of the study. The main macroscopic lesions and mean lesions scores are shown in Table 1 and Table 2.

### 3.2. Fecal Shedding, Viral Load, and Tissue Distribution in PAstV-Inoculated Pigs

**Figure 2:**
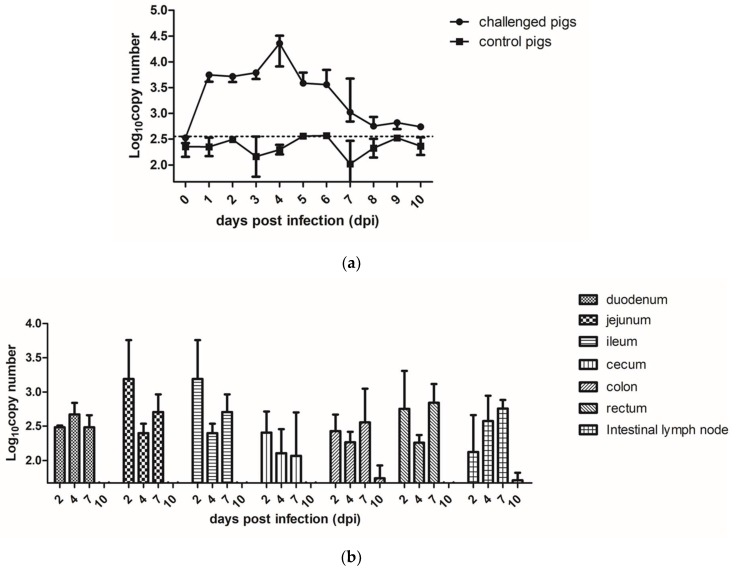
The RNA copy numbers of PAstV in different samples. (**a**) The fecal PAstV shedding in challenged and control pigs at days post inoculation (dpi). (**b**) The PAstV viral RNA load in different intestinal segments at different days post challenge. Mean ± SD (error bars) viral load are shown.

During the process of the animal experiments (10 days), fecal shedding of the PAstV-challenged (3 pigs) and control groups (3 pigs) were detected by qRT-PCR, as shown in Figure 2a. PAstV RNA in fecal samples was detected from all the three piglets in the PAstV-challenged group at 1 dpi. All the PAstV-challenged piglets shed PAstV in their feces between 1 to 6 dpi. The viral shedding in the feces diminished thereafter until 10 dpi.

The levels of PAstV RNA in different organs at the indicated time points were evaluated by qRT-PCR. The results showed that the PAstV RNA could be detected in the intestines and lymph nodes at 2 dpi, with the jejunum and ileum segments in PAstV-challenged group reaching the highest level. The number of PAstV RNA copies gradually declined at 4 dpi and 7 dpi, and reached the same level of the control group at 10 dpi. (Figure 2b). We also detected the RNA of PAstV in the spleen, lungs, kidneys, and brain, but the specific pathogen particles could not be detected in these organs.

### 3.3. Histopathological and Morphometric Changes Induced by PAstV1-GX1 Infection

**Figure 3:**
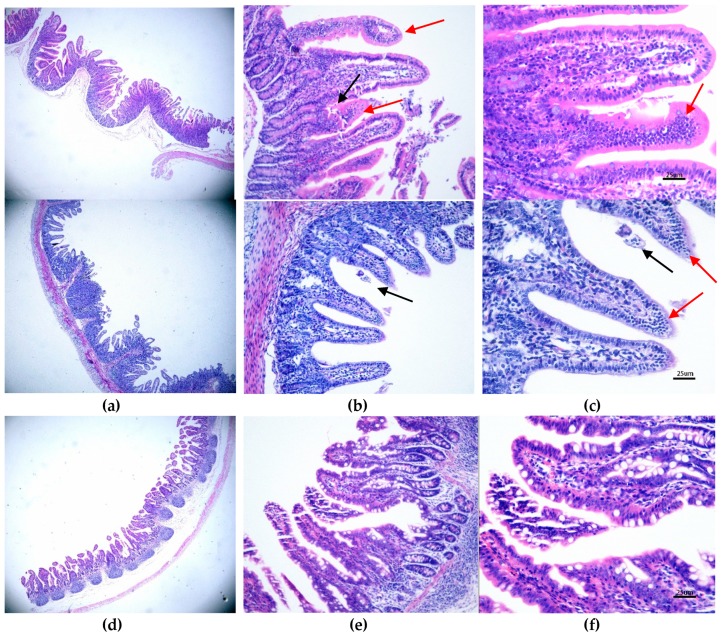

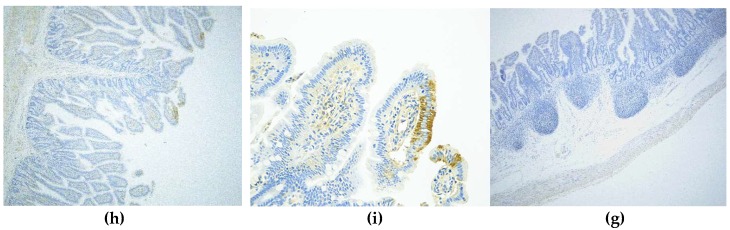
The microscopic lesions changed at different days and the IHC staining results of PAstV-challenged piglet intestines. HE staining of a section from a PAstV-challenged piglet jejunum at 2 dpi at (**a**) 4×, (**b**) 20×, and (**c**) 40× magnification. (c) The black arrow indicates villous damage, while the red arrow indicates that many neutrophils and macrophages were seen in the tip of villi. The control group under the same condition are shown at (**d**) 4×, (**e**) 20×, and (**f**) 40× magnification. For IHC, the jejunum sections at 4 dpi were stained with a PAstV capsid polyclonal antibody (1:200 dilution) at (**h**) 10× and (**i**) 40× magnification. (**g**) The control group under 10× magnification is also shown.

The control group exhibited normal intestinal histopathology (Figures 3c and 5f). In the PAstV-challenged group, the histopathologic lesions were mainly observed in the duodenum, jejunum, ileum, and mesenteric lymph nodes. At 2 and 4 dpi, many neutrophils were seen in the tip of villi of the small intestinal, and mild damage to some of villi of the small intestinal was found (Figure 3b,c). Macrophages and villi vasodilatation were also observed (Figure 3a,b). Crypt hyperplasia and villi atrophy and fusion were pronounced, but most of the above lesions gradually recovered by 7 and 10 dpi. No discernible microscopic lesions were observed in cecum or colonic sections of PAstV-challenged pigs for the duration of the study.

The intestinal villous height of the control and the PAstV-challenged piglets were measured at the indicated day (2, 4, 7, and 10 dpi). As shown in Table 3, the villous height of the duodenum and jejunum from challenged piglets significantly decreased at 2 dpi compared with the control group. At 4 dpi, the villous height of all three intestinal sections from challenged pigs (duodenum: 274.1 ± 16.22 μm, jejunum: 260.9 ± 14.73 μm, and ileum: 264 ± 14.76 μm) significantly decreased compared to the control group (duodenum: 513.9 ± 68.77 μm, jejunum: 445.4 ± 47.62 μm, and ileum: 333.7 ± 11.59 μm) (*p* < 0.001). However, the villous heights of the intestine of challenged pigs were not significantly different with those of control pigs at 7 and 10 dpi.

The crypt depth of challenged and control pigs were also surveyed at 2, 4, 7, and 10 dpi. The results showed that at 2 dpi, the crypt depths of the duodenum, jejunum, and ileum from challenged pigs (180.7 ± 13.21, 171.5 ± 9.24, and 179.5 ± 22.36, respectively) were significantly higher than those of the control groups (147.1 ± 8.64, 147 ± 4.97, and 121.6 ± 6.5, respectively; *p* < 0.05). However, at the other time points, the crypt depths of the intestine were not significantly different between challenged and control pigs (Table 3). 

The ratios of villous height and crypt depth (VH/CD ratios) for the control and challenged groups at each time point are shown in Table 3. The mean ratios of the challenged pigs were significantly lower than the control pigs at 2 and 4 dpi (*p* < 0.05). However, at 7 and 10 dpi, they were not significantly different.

The PAstV antigen appeared in the cytoplasm of villous enterocytes at 2, 4, 7, and 10 dpi in the challenged pigs. The antigen labeling was brownish-yellow granules with diffuse distribution, and was not found in the nucleus (Figure 3h,i). Strong antigen labeling was observed in the duodenum, jejunum, ileum, and intestinal lymph nodes at 2 dpi, with medium labeling at 4 and 7 dpi and mild labeling at 10 dpi. The cecum, colon, and rectum showed only mild antigenic markers until 7 dpi, and no labeling at 10 dpi (Table 4). The results of semiquantitative immunohistochemistry assay in different intestinal segments are shown in Table 4. Strong antigen labeling (IRS > 7) was observed in the jejunum, ileum, and intestinal lymph nodes at 2 dpi and in the duodenum at 4 dpi, with moderate positive staining (4 < IRS < 6) in the duodenum at 2 dpi and in the duodenum and jejunum at 7 dpi, along with either mild or negative staining at 10 dpi. The cecum, colon. and rectum showed mild antigenic positive staining (2 < IRS < 4) until 4 dpi, and negative staining at 7 or 10 dpi.

### 3.4. The Effects of PAstV on Transcriptional Activation of Inflammatory Cytokines and Permeable Proteins

In order to investigate the innate immune response genes which were induced in piglets infected with PAstVs, qRT-PCR was performed with RNA samples from different intestinal segments in PAstV-challenged or control piglets. The results showed the mRNA levels of IFNβ significantly increased in the jejunum, ileum, cecum, and colon at 2 dpi, and in the duodenum, jejunum, ileum, and rectum at 4 dpi, respectively. The IFNβ expression levels declined gradually by 7 dpi. On the other hand, the mRNA expression levels of ISG54 were slightly upregulated in the jejunum, ileum, and colon at 2 dpi and in the duodenum and rectum at 4 dpi. No significant differences of expression of IFNβ and ISG54 were observed in PAstV-challenged and control pigs at 7 dpi. Interestingly, the mRNA expression levels of type III interferon (IFN Lambda 1, IFNλ1) were significantly downregulated in each segment of the intestine at 2–7 dpi. No signification changes of these three genes were observed in all intestinal segments in control groups at all different time points.

**Figure 4:**
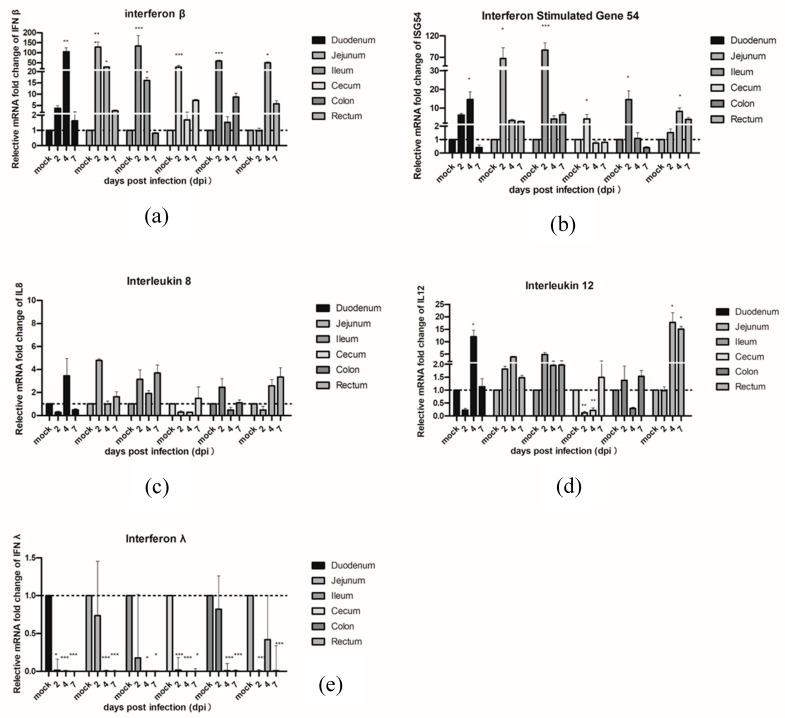
Quantification of the mRNA expression levels of (**a**) IFNβ and (**b**) ISG54 and pro-inflammatory cytokines (**c**) IL8, (**d**) IL12, and (**e**) IFNλ1 by qRT-PCR in PAstV-challenged piglets at different days after infection. The qRT-PCR values with primers (Table S1) for different cytokines were normalized with endogenous β-actin mRNA levels on each day post infection. The fold change of relative gene expression levels was calculated using the using the formula: 2 − (ΔCt of gene − ΔCt of β-actin). Data represent the average of two independent samples from each piglet in the PAstV-challenged group and each sample was measured in triplicate. Error bars indicate standard deviations (SD). Asterisks show statistical significance as measured by the two-tailed Student t test, as follows: *, **, and *** represent *p* < 0.05, *p* < 0.01, and *p* < 0.001, respectively.

In terms of pro-inflammatory cytokines, IL8, and IL12, PAstV-challenged pigs showed higher mRNA expression of IL12 than the control group pigs in the duodenum and rectum at 4 dpi and in the rectum at 7 dpi, respectively. In addition, the IL12 mRNA was downregulated at 2 and 4 dpi in the cecum. There were also no significant changes observed in control groups at all the different time points tested (Figure 4).

**Figure 5:**
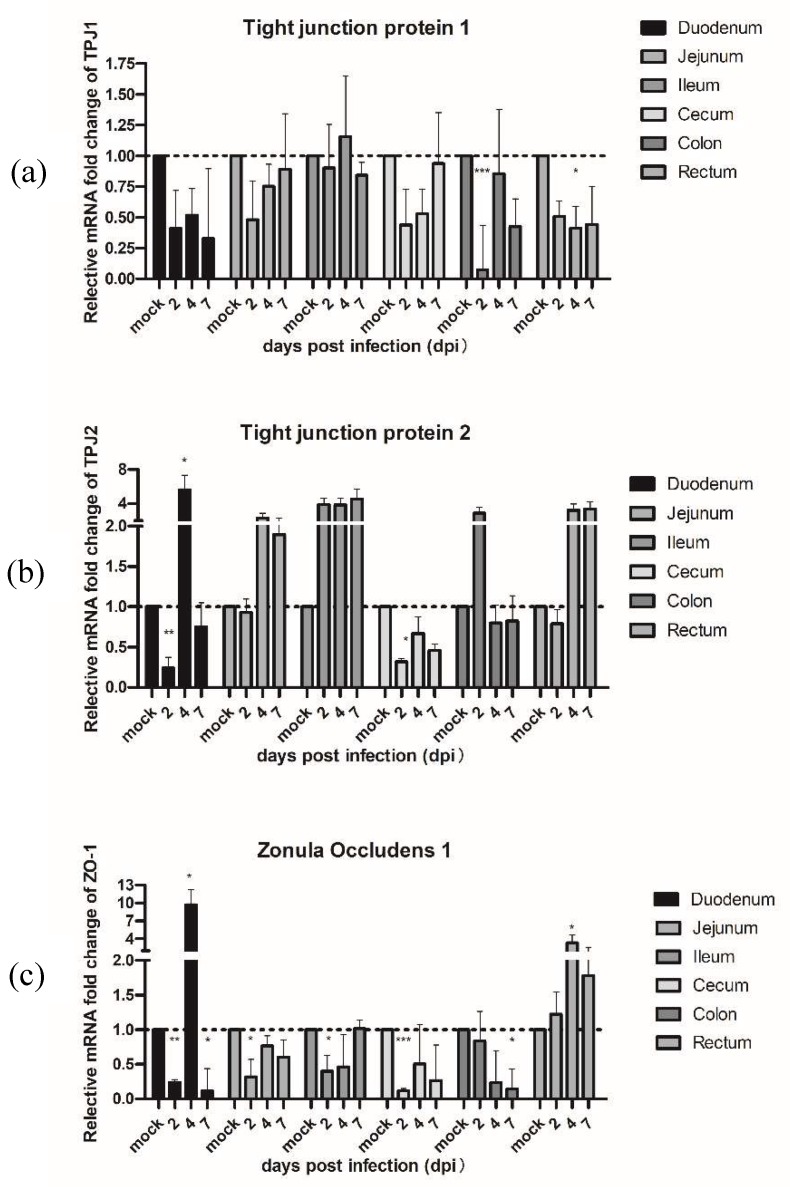
Quantification of the mRNA expression levels of (**a**) tight junction protein 1 and (**b**) 2 and (**c**) zonula occludin 1 (ZO-1) by qRT-PCR in PAstV-challenged piglets at different days after infection. The qRT-PCR values with primers (Table S1) for different cytokines were normalized with endogenous β-actin mRNA levels on each day post infection. The fold change of relative gene expression levels was calculated using the formula: 2 − (ΔCt of gene − ΔCt of β-actin). Data represent the average of two independent samples from each piglet in the PAstV-challenged group and each sample was measured in triplicate. Error bars indicate standard deviations (SD). Asterisks show statistical significance as measured by the two-tailed Student t test as follows: *, **, and *** represent *p* < 0.05, *p* < 0.01, and *p* < 0.001, respectively.

To confirm the hypothesis that PAstV also could affect the expression of some proteins associated with the intestinal permeability barrier, we measured the mRNA levels of tight junction protein 1 (TJP1) and 2 (TJP2) and zonula occludin 1 (ZO-1) by qRT-PCR (Figure 5). The results demonstrated that the mRNA expression of TJP1 in challenged pigs was significantly downregulated in the colon at 2 dpi and in the rectum at 4 dpi, respectively. The mRNA of TJP2 in PAstV-challenged pigs was significantly downregulated in the duodenum and cecum at 2 dpi, and then rebounded by 4 dpi. In addition, significant decreases were also observed in ZO-1 at 2 dpi, except for the colon and rectum, but mRNA expression of ZO-1 in the duodenum, cecum, and colon were still lower than the control at 7 dpi.

## 4. Discussion

PAstV is widespread and highly prevalent all over the world, including in the United States [6], Canada [32], Croatia [4], Italy [33], Germany [34], Thailand [35], South Korea [36], and China [5,37]. Most studies in the field of PAstV have only focused on the study of molecular characterization and epidemiology. It is generally assumed that PAstV is an enteropathogenic virus and can cause diarrhea in piglets, but there is a lack of related pathogenesis evidence to support this view. In the present study, we inoculated 7-day-old piglets with a porcine astrovirus strain (PAstV1-GX1) to investigate the pathogenicity of PAstV. By comparing the clinical signs, average daily gain (ADG), macroscopic and microscopic lesions, magnitude and duration of RNA titers in the feces and organs, immunohistochemistry, and morphometry of the villous height and crypt depth between PAstV-challenged and control piglets, we showed that PastV infection could damage the intestinal tract and cause growth retardation. In addition, PAstV infection upregulated the expression of INFβ and ISG54. Interestingly, the mRNA expression of TJP1 and ZO-1, which are associated with intestinal permeability, were significantly decreased after PAstV infection, suggesting that intestinal damage induced by the virus was related to an increase in the intestinal permeability barrier.

Generally, AstVs are considered to be enteroviruses, causing diarrhea in several mammalian species [3,38,39,40]. In particular, human astrovirues (HAstVs) have been recognized as the second most common cause of viral diarrhea in young children [1]. HAstV infections can cause mild and watery diarrhea, and individual cases can also lead to vomiting, fever, anorexia, and abdominal pain. Most of these symptoms would recover without medicine intervention [41]. However, a large proportion of astroviral infections can be asymptomatic [2,13,42]. Turkeys and mice are the only two kinds of animal models used to study the pathogenesis of TuAstV and MuAstV, respectively [43,44]. Our present study shows that PAstV-challenged pigs displayed decreased appetite, irregular hair coat, and most of pigs had semisolid feces at 2 dpi. The main symptoms of PAstV-challenged pigs were flatulence, gastric capillary and mesenteric congestion, and macroscopic lesions in the intestine containing large amounts of yellow foamy liquid. These lesions were observed at 2 dpi and became more conspicuous at 4 dpi, but were recovered by 10 dpi. These results suggest that PAstV infection can cause intestinal damage in piglets, but this damage is usually recovered within 10 days.

Our study also showed that PAstV infection could affect the ADWG in piglets. A significantly reduction (*p* < 0.01) in ADWG of PAstV-challenged pigs was observed at 7 dpi. The reasons for the decline of ADWG might be due to the intestinal damage observed and to decreased appetite. With the gradual recovery of intestinal damaged, the ADWG of PAstV-challenged pigs returned to normal by 10 dpi. Notably, the total body weight of PAstV-challenged pigs could not compensate for the initial loss during this study, indicating that PAstV-challenged pigs may be reared 2–3 days longer than normal pigs in order for these animals to reach the market.

Previous studies showed the TAstV-2 could be detected in different organs, including serum, and this caused enteric infection and decreased thymus size in infected turkeys [43]. Yokoyama et al. reported the viral genome copies of MuAstV could be detected in different tissues in mice [44]. Our study also showed the RNA of PAstV could be detected at 2 dpi and reached a peak at 4 dpi in the duodenum, jejunum, and ileum, and this began to wane at 7 to 10 dpi. The results of microscopic histology and morphometry showed that the main lesions were the many neutrophils that infiltrated at the top of the villi in the small intestine and caused damage to some of the villi of the small intestine, accompanied by lymphocyte and neutrophil infiltration at 4 dpi. By 10 dpi, the lesions were recovered. It was suggested that PAstV infection mainly damaged the villous epithelium and caused a mild inflammatory reaction in intestinal and mesenteric lymph nodes.

Recently, many researchers found AstVs could cause extragastrointestinal infection, and this was associated with encephalitis and meningitis [45]. By using unbiased pyrosequencing techniques, Quan et al. reported that HAstV was the most probable causative agent responsible for encephalitis in a 15-year-old boy with agamma-globulinemia [46]. The perineural invasion and viremia seen was also observed in other animal astroviruses, such as cattle [47,48], sheep [49], pig [20], and turkey [43]. In this study, no PAstVs were detected in the spleen, lungs, kidneys, and brain, which suggested that different genotypes of PAstVs may have different forms of cell tropism.

Little is known about the innate immune response to astrovirus infection in pigs. Shauna et al. showed that type I interferon limits replication of human astrovirus type 1 and mouse astrovirus type STL, both in vitro and in vivo [50]. Yokoyama et al. demonstrated that adaptive immunity is required to control MuAstV infection [44]. However, HAstVs induced a mild and delayed IFN response in CaCo-2 cells [51]. In this study, we found the expression levels of IFNβ and ISG54 were upregulated in the ileum at 2–4 dpi, suggesting that type I interferon may play an important role in host anti-PAstV infection. The expression levels of IFNλ in the intestine were significantly downregulated. IFNλ is a new class of interferons that have similar functions acting to inhibit viral replication, similar to type I interferon [52]. The mechanism of PAstV in reducing the expression of IFNλ is unclear and needs further study.

Recently, a number of studies have suggested that astroviruses induced diarrhea by increasing the intestinal barrier permeability [50,53,54]. We also observed decreases in the expression of TJP1, TJP2, and ZO-1 at 2 dpi in all segments of the intestine, and the ZO-1 was affected more markedly than TJP1 and TJP2 in PAstV-challenged animals. These results imply that PAstV infection might damage expression of proteins associated with the permeability barrier.

## 5. Conclusions

To summarize, the pathogenesis of PAstV in seven-day-old nursing piglets was studied using PAstV1-GX1. PAstV1-GX1 infection could cause mild diarrhea, growth retardation, and damage of the villi of the small intestinal mucosa. However, all the above symptoms could be restored within 7 to 10 dpi. The mRNA expression levels of IFNβ and ISG54 were significantly elevated in PAstV-challenged piglets. In contrast, expression of IFNλ was downregulated in piglets infected with PAstV1-GX1. The mRNA expression of TJP1, TJP2, and ZO-1, which are associated with the intestinal permeability barrier, were affected by the PAstV1 infection.

## Figures and Tables

**Table 1:** The macroscopic characteristic lesions at given days post inoculation (dpi).

dpi	PAstV-Challenged	Control
2 d	Stomach bloat, gastric capillary congestion, small intestine bloat containing yellow foam liquid, mesenteric congestion	Mildly flatulence in individual pigs of the small intestine
4 d	Stomach bloat, gastric capillary congestion, small intestine bloat containing yellow foam liquid, mesenteric congestion, cecum slightly flatulent	No lesions
7 d	Yellow liquid contained in individual pigs of the small intestine	No lesions
10 d	No lesions	No lesions

**Table 2:** The mean scores of the macroscopic lesions from different intestinal segments.

	PAstV-Challenged	Control
Duodenum	0.42 ± 0.19	0
Jejunum	1.17 ± 0.24	0.73 ± 0.20
Ileum	0.83 ± 0.21	0.36 ± 0.15
cecum	0.17 ± 0.11	0
colon	0.08 ± 0.08	0.09 ± 0.09
rectum	0	0
mesenteric lymph	0.5 ± 0.15	0
spleen	0	0
ung	0	0
kidney	0	0
stomach	0.5 ± 0.15	0.27 ± 0.14

**Table 3:** Mean villous height, crypt depth, and villous height to crypt depth ratios in different segments at given days post inoculation (dpi).

dpi		Duodenum			Jejunum			Ileum		
		Control	Challenged	*p*	Control	Challenged	*p*	Control	Challenged	*p*
2	Villous height (μm)	544.9 ± 15.88	447.7 ± 25.82	0.038 (*)	461.6 ± 27.97	268.1 ± 27.97	0.0001 (***)	344.9 ± 47.67	278.6 ± 47.67	0.2328 (NS)
	Crypt depth (μm)	147.1 ± 8.644	180.7 ± 13.21	0.0328 (*)	147.0 ± 4.974	171.5 ± 9.240	0.0161 (*)	121.6 ± 6.506	179.5 ± 22.36	0.002 (*)
	Ratio (μm/μm)	3.704 ± 1.828	2.478 ± 1.955		3.140 ± 5.623	1.563 ± 0.0003		2.836 ± 7.327	1.558 ± 1.21	
4	Villous height (μm)	513.9 ± 68.77	274.1 ± 16.22	0.0003 (***)	445.4 ± 47.62	260.9 ± 14.73	0.0003 (***)	333.7 ± 11.59	264 ± 14.76	0.0001 (***)
	Crypt depth (μm)	168.3 ± 5.602	190.4 ± 11.12	0.1936 (NS)	162.6 ± 7.897	161.3 ± 6.637	0.917 (NS)	122.5 ± 6.575	145.6 ± 11.06	0.0649 (NS)
	Ratio (μm/μm)	3.053 ± 12.276	1.440 ± 1.459		2.739 ± 6.03	1.617 ± 2.219		2.734 ± 1.763	1.813 ± 1.335	
7	Villous height (μm)	456.5 ± 32.36	420.1 ± 24.66	0.4361 (NS)	442.3 ± 29.53	451.6 ± 40.22	0.8539 (NS)	380 ± 22.29	310.2 ± 22.29	0.0728 (NS)
	Crypt depth (μm)	176.7 ± 5.904	182.0 ± 14.98	0.8042 (NS)	162.6 ± 15.35	177.6 ± 11.22	0.4573 (NS)	157.3 ± 6.014	138.8 ± 11.52	0.1380 (NS)
	Ratio (μm/μm)	2.583 ± 5.481	2.308 ± 1.646		2.720 ± 1.924	2.543 ± 3.585		2.416 ± 3.706	2.235 ± 1.935	
10	Villous height (μm)	510.1 ± 9.831	485.4 ± 10.76	0.1181 (NS)	312.6 ± 16.88	336 ± 17.5	0.3513 (NS)	394.9 ± 8.937	367.8 ± 21.71	0.3243 (NS)
	Crypt depth (μm)	188.4 ± 14.14	211.7 ± 14.12	0.5219 (NS)	194.3 ± 7.024	192.4 ± 8.213	0.8751 (NS)	158 ± 16.27	162.6 ± 8.119	0.7906 (NS)
	Ratio (μm/μm)	2.571 ± 0.695	2.293 ± 0.762		1.609 ± 2.403	1.746 ± 2.131		2.429 ± 0.549	2.262 ± 2.674	

* represent statistically significant. *** represent statistically very significant. NS represent difference with no significance.

**Table 4:** The immunoreactive scores of the PAstV immunohistochemistry from challenged pigs as determined by days post inoculation (dpi).

dpi	Number of Sections	Duodenum	Jejunum	Ileum	Cecum	Colon	Rectum	Mesenteric Lymph
2	3	6.67 ± 0.67	8.33 ± 2.33	9.00 ± 1.73	1.67 ± 0.33	2.00 ± 1.00	2.00 ± 1.00	7.33 ± 1.67
4	4	8.50 ± 1.66	6.25 ± 1.32	3.25 ± 0.75	1.25 ± 0.25	2.25 ± 1.32	1.13 ± 0.48	2.00 ± 1.35
7	3	5.00 ± 2.08	4.67 ± 1.33	2.33 ± 0.67	2.33 ± 0.88	1.67 ± 0.88	1.33 ± 0.67	1.33 ± 0.33
10	3	4.00 ± 2.65	2.00 ± 1.16	1.67 ± 1.20	1.00 ± 0.58	0.67 ± 0.33	0.67 ± 0.33	0.67 ± 0.33

## Supplementary Materials

The following are available online at https://www.mdpi.com/1999-4915/11/12/1156/s1. Table S1: Primers used in this study.
