# Supplementary material for: Pathogenic Characteristics of a Porcine Astrovirus Strain Isolated in China
Source: Viruses. 2019 Dec 13;11(12):1156. doi: 10.3390/v11121156 (PMC6949928; doi:10.3390/v11121156)
Supplement: Supplementary file 1 [file viruses-11-01156-s001.pdf]

Table S1: Primers used in this study

Primer name	Primer sequence 5'-3'	Reference
PAstV 1F	TCCTGTGCTATCAGTTGCTCTC	GO914773
PAstV 1R	GATTGCTGGTTTTGGACCTGTG	
PAstV 2F	AGCAGCTGGATCGTCTTTGGA	JX556690
PAstV 2R	AGATTCAGCATCCCAGGTTGTT	
PAstV 4F	TGGCTTCAGGCCTTTGAGTTTT	JX556692
PAstV 4R	CACCGTCGTAGTAGTCGTGAC	
PAstV 5F	TGGTACGTRCACAATCTGTTGAA	JX556693
PAstV5R	TCAGTGTCTTCCCAACCRTC	
RV-P1	AAATCCGCAACTATACTGTGTGACTA	FJ807867
RV-P2	TGGCCAACTGGTTCTGTCTA	
TGEV-P3	CAACCCTGAAACTAACGCAATTCT	FJ755618
TGEV-P4	GCCCATCCAGTCGCACTACTT	
PEDV-P5	AGGAACGTGACCTYAAAGACATCCC	AF353511
PEDV-P6	GCCCATCCAGTCGCACTACTT	
PAstV-qF	TGGCAAAGATGTCACTGTCAAGGTC	NC_025379
PAstV-qR	CGCTCCTGGTTTAGAGTTGATGGTA	
AstV-M1F	GCT ATA CCG GTG GTG CTG TTA T	
AstV-M1R	GAG TTC CTT GTT TGG GCC ATT	
AstV-M2F	CACTCTGCGCCATTGAGAC	
AstV-M2R	TCTCCACCTCGGCTTCTAAG	
AstV-M3F	CAACCAACCCGGATGATGAT	
AstV-M3R	GACATTCCGTCCTGCGTCTT	
TJP1-O3	TTTTCCTACTTGACCTCCCG	XM_021098833
TJP1-O4	ATCTCCCACATCCGACTCTA	
TJP2-O3	ATCTCCCACATCCGACTCTA	
TJP2-O4	ATCTCCCACATCCGACTCTA	
ZO-1 O3	GCCGCCTCCTGAGTTTGA	AU318101
ZO-1 O4	ACCCCGCCGTTGCTGTTA	
IL-8 F	CCGTGTCAACATGACTTCCAA	MM_2138671
IL-8 R	GCCTCACAGAGAGCTGCAGAA	
IL-12 F	AACTCTTCACGGACCAAATCTCA	MM_2138671
IL-12 R	GGTCCCGGGCTTGCA	
IFN-β F	GCTCTCCTGATGTGTTTCTC	KJ147517
IFN-β R	AGGGACCTCAAAGTTCATCC	
ISG54 F	AAGAACTCCTTGGAGAGCTG	JX070559
ISG54 R	CCTGTATGTTGCACATCGTG	
β-actin F	CTCCATCATGAAGTGCGACGT	U07786
β-actin R	GTGATCTCCTTCTGCATCCTGTC	
